# The β2-adrenergic receptor antagonist ICI-118,551 blocks the constitutively activated HIF signalling in hemangioblastomas from von Hippel-Lindau disease

**DOI:** 10.1038/s41598-019-46448-6

**Published:** 2019-07-11

**Authors:** A. M. Cuesta, V. Albiñana, E. Gallardo-Vara, L. Recio-Poveda, I. de Rojas-P, K. Villar Gómez de Las Heras, D. T. Aguirre, L. M. Botella

**Affiliations:** 1 Centro de Investigaciones Biológicas, Consejo Superior de Investigaciones Científicas (CSIC), and Centro de Investigación Biomédica en Red de Enfermedades Raras (CIBERER), group U707, 28040 Madrid, Spain; 2 SSCC del Servicio de Salud de Castilla-La Mancha (SESCAM), Toledo, Spain; 3 Department of Neurosurgery, Fundación Jiménez Díaz (IIS-FJD), Av. Reyes Católicos, 28040 Madrid, Spain

**Keywords:** Growth factor signalling, Growth factor signalling, Molecular medicine, Molecular medicine

## Abstract

One of the major consequences of the lack of a functional VHL protein in von Hippel-Lindau disease, a rare cancer, is the constitutive activation of the HIF pathway. This activation ends up in the generation of Central Nervous System (CNS) Hemangioblastomas among other tumours along the lifespan of the patient. Nowadays, only surgery has been proven efficient as therapy since the systemic attempts have failed. Propranolol, a non-specific β1-and β2-adrenergic receptor antagonist, was recently designated as the first therapeutic (orphan) drug for VHL disease. Nevertheless, its β1 affinity provokes the decrease in blood pressure, being not recommended for low or regular blood pressure VHL patients. In order to overcome the β1-drawback, the properties of a high specific β2-adrenergic receptor blocker named ICI-118,551 have been studied. ICI-118,551 was able to decrease Hemangioblastomas cell viability in a specific manner, by triggering apoptosis. Moreover, ICI-118,551 also impaired the nuclear internalization of HIF-1α in Hemangioblastomas and hypoxic primary endothelial cells, reducing significantly the activation of HIF-target genes and halting the tumour-related angiogenic processes. In this work, we demonstrate the therapeutical properties of ICI-118,551 in VHL-derived CNS-Hemangioblastoma primary cultures, becoming a promising drug for VHL disease and other HIF-related diseases.

## Introduction

Von Hippel-Lindau (VHL) disease is an autosomal dominant inherited genetic disorder with an incidence of 1 per 36,000 individuals in the general population and is considered as a rare disease or rare cancer^1,2^.

Patients with VHL disease harbour a single mutation allele in the tumour suppressor gene *VHL* (3p25–p26). VHL protein (pVHL) controls the cytoplasmic levels of the Hypoxia Inducible Factor (HIF) complex. In normoxic conditions, pVHL binds to the previously hydroxylated prolyl residues of HIF-1α and HIF-2α, being ubiquitinated and targeted to the proteasome for rapid degradation. When patients suffer a spontaneous inactivation or loss of the second wild-type VHL allele (loss of heterozygosity), either there is no expression of the pVHL or the mutated form is not functional^3,4^.

Therefore, in the absence of functional pVHL, HIF-1α and HIF-2α subunits accumulate within the cytoplasm and translocate to the nucleus, triggering the hypoxia program by targeting hypoxia responsive genes, which are normally silenced in normoxia^5^. HIF-1α and HIF-2α are involved in cell proliferation, angiogenesis, extracellular matrix degradation, vascular tone, and erythropoiesis, among other processes. Hence, cells from VHL tumours have a constitutively active HIF program (a pseudo-hypoxic state) due to the absence of functional pVHL^6,7^.

As a consequence of this pseudo-hypoxic state, the clinical manifestations of the disease include multiple benign and malignant tumours that appear throughout the lifespan of the patient: retinal hemangioblastoma, Central Nervous System (CNS) hemangioblastoma, clear cell renal cell carcinoma (ccRCC), pheochromocytoma, pancreatic islet tumour, endolymphatic sac tumours, and cysts in testes and broad ligament^8–11^.

Thus far, therapeutic options for VHL patients are only derived from surgery and, unfortunately, the preoperative neurological deficits do not resolve after surgery^12^. In addition, the systemic therapies, mainly antiangiogenic, used for metastatic cancers had shown limited response in VHL tumours^13,14^. Therefore, and due to the lack of effective therapies for diffuse or recurrent disease, there is an urgent demand for effective drugs for VHL patients, especially those medicines that might halt the progression of tumours and subsequently delay surgical treatment.

In 2008, Léauté-Labrèze and col. showed for the first time the therapeutical antiangiogenic properties of Propranolol, a non-specific β1-and β2-blocker with more than fifty years on the market. Propranolol is prescribed for the treatment of arrhythmia, hypertension, migraines, and other cardiac and neurological diseases, as well as infantile hemangioma^15–18^. In addition, our lab has demonstrated *in vitro* and in a clinical trial (EudraCT: 2014-003671-30), the therapeutical properties of Propranolol in VHL disease^19,20^. In brief, Propranolol-treated primary cultured hemangioblastoma (HB) cells decreased the RNA and protein expression levels of HIF-1α and HIF-2α, and therefore the pro-angiogenic and HIF-1α targets such us *Endoglin*, *VEGF*, *EPO*, and *SOX*. Moreover, *VEGF* and miR210 plasma levels from treated patients were reduced after treatment with Propranolol^19,21^.

Remarkably, the main property of Propranolol, its capacity of reduce the blood pressure, is the highest drawback for low or regular blood pressure VHL patients. Alongside the clinical trial, hypotension and bradycardia were the main side effects registered. These side effects of Propranolol are driven exclusively by its β1-adrenergic receptor blockade^22^, so it would be possible to overtake them and keep the benefits by using a high specific β2-adrenergic receptor antagonist.

In this line, β2-blockers have shown antiangiogenic properties *in vitro* and *in vivo*^23–25^, becoming a promising therapeutical molecule for VHL Hemangioblastomas^26^. Among them lays the selective antagonist of the β2-adrenergic receptor erythro-D,L-1(methylinden-4-yloxy)-3-isopropylaminobutan-2-ol, known as ICI-118,551 (ICI).

ICI was developed by Imperial Chemical Industries in the 80’s. Up to now, ICI has no known therapeutic application in humans so far, although it has been used widely in research to understand the action of the β2 adrenergic receptor due to its β2/β1 selectivity ratio of 123 folds and a Kd in nM range^27–33^. Several human clinical studies as well as murine/rodent models developed in the 80’s used ICI in comparative analysis of different β-antagonists. Supplementary Table 1 shows a compilation of the assays performed with ICI in humans, including purpose, results, dose, and length of the treatment. Nevertheless, little is known about ICI’s pharmacokinetics; it is known that ICI is able to cross the blood–brain barrier (BBB)^34–36^ (Supplementary Table 1). Based on the clinical trials and *in vivo* assays done, systemic doses have been used without toxicity in humans (up to 20 mg/kg), rodents (0.5 or 1 mg/kg) or rhesus monkeys (0.1 μg/kg). ICI, nowadays, is mainly used as control of β-blockers’s specificity in physiological processes such us control of heart and vascular tone and SNC and muscular signaling^37–40^. Finally, ICI has been also used in cancer research showing a role in cell proliferation and signalling regulation^41,42^.

Here we show a future therapeutic approach of ICI for VHL disease and a potential mechanism of action using different HB primary cultures. Blockage of the β2-receptor impairs the viability of HBs primary culture cells by triggering the apoptotic caspases 3 and 7 cascade. Moreover, ICI is able to halt or almost delay two main angiogenic processes such us cell migration and tube formation. Finally, and based on the idea that β-antagonists downregulate HIF signalling, we demonstrate that ICI prevents the nuclear internalization of HIF-1α in HBs and human endothelial primary cultures under hypoxic conditions.

Taken together the above mentioned effects on VHL hemangioblastomas plus the lack of side-off effects on healthy endothelial cells and blood pressure^43^, ICI arises as a promising new (and almost unique) molecule on the field of VHL therapies. Its properties suggest also a potential therapeutic role on other angiogenesis-related diseases like Hereditary Hemorrhagic Telangiectasia (HHT) or carcinomas.

## Results

### ICI-118,551 decreases viability in hemangioblastomas by inducing cell apoptosis

**Figure 1:**
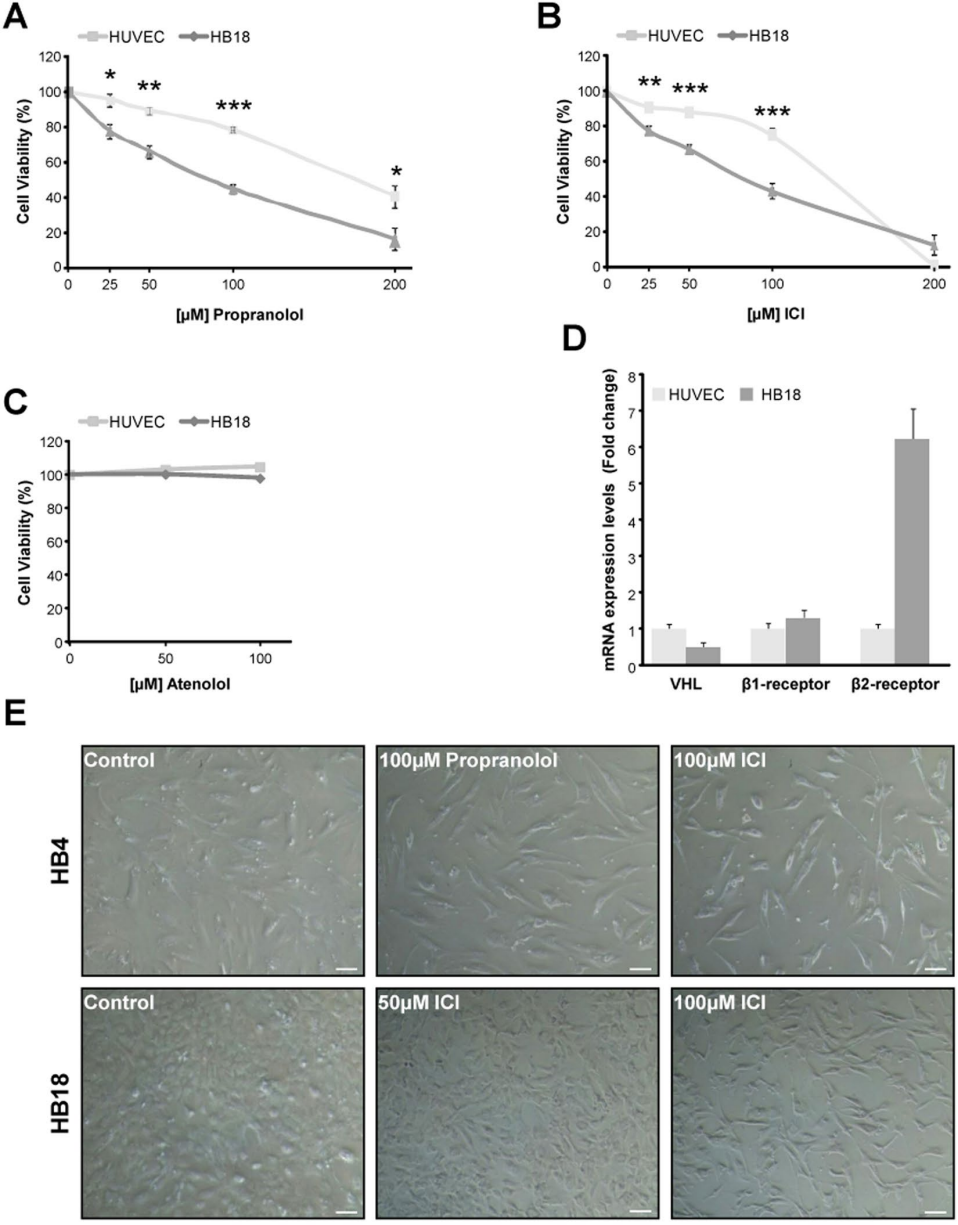
Effect of β2-adrenergic receptor blockage on HB cells viability. HB (dark grey) and HUVEC (light grey) primary cultures were incubated for 48 hours with increasing doses [0–100/200 µM] Propranolol (**A**), ICI (**B**), and Atenolol. (**C**) Propranolol and ICI significantly impair cell viability only in HB cells, while Atenolol showed no changes in cell viability neither in HBs nor in HUVECs. Cell viability was measured as described in Material and Methods. (**D**) mRNA quantification of β1-and β2-receptors in HB cells versus HUVECs by RT-qPCR. Data show a higher expression of β1-and β2-receptors in HBs compared with HUVECs. (**E**) Representative pictures of 8 HB primary cell cultures treated with 50–100 µM Propranolol or ICI for 96 hours. Data denote mean ± S.D (n = 3). Scale bars represent 100 µm.

The selective β2-receptor blocker ICI mirrors the results previously obtained with the non-selective β1-and β2-antagonist Propranolol^21^. When testing viability of HB cells in a dose-response curve Propranolol (Fig. 1A) and ICI (Fig. 1B) decreased viability of primary cultures of VHL HB cells in a dose-dependent manner. Our lab had previously established 100 μM Propranolol as the effective dose; this dose is able to impair cell viability at least by 55–60% when compared to untreated cells, and ICI gives similar results. Dose-effect curves for ICI and Propranolol are also shown for HUVECs (Fig. 1C). Interestingly, the effect of the β-blockers seems to be rather specific for HB cells, since at 100 μM there is a significant difference of viability, while in HUVECs is of 80%, in HB drops to 40–45%.

In order to determine the β2-receptor specificity, Atenolol, a specific β1-receptor antagonist, was used to test for the effect on viability at 50 and 100 μM (Fig. 1C). Atenolol, in comparison with ICI (Fig. 1B) and Propranolol (Fig. 1A), hardly provokes any effect on HB and HUVECs.

As the difference in viability between HUVECs and HB cells could be explained by the number of β-receptors expressed, we then measured the gene expression levels by RT-qPCR of β1 and β2 receptors. As it is shown in Fig. 1D, the amount of both β-receptors and more specific the levels of β2 receptors were higher in HB than HUVECs.

Figure 1E shows the cells remaining in HB cultures from 2 different patients after 72 hours of treatment with 100 μM Propranolol and 50 and 100 μM ICI compared with untreated cells. A significant visual reduction in the number of cells with respect to the untreated ones is observed.

**Figure 2:**
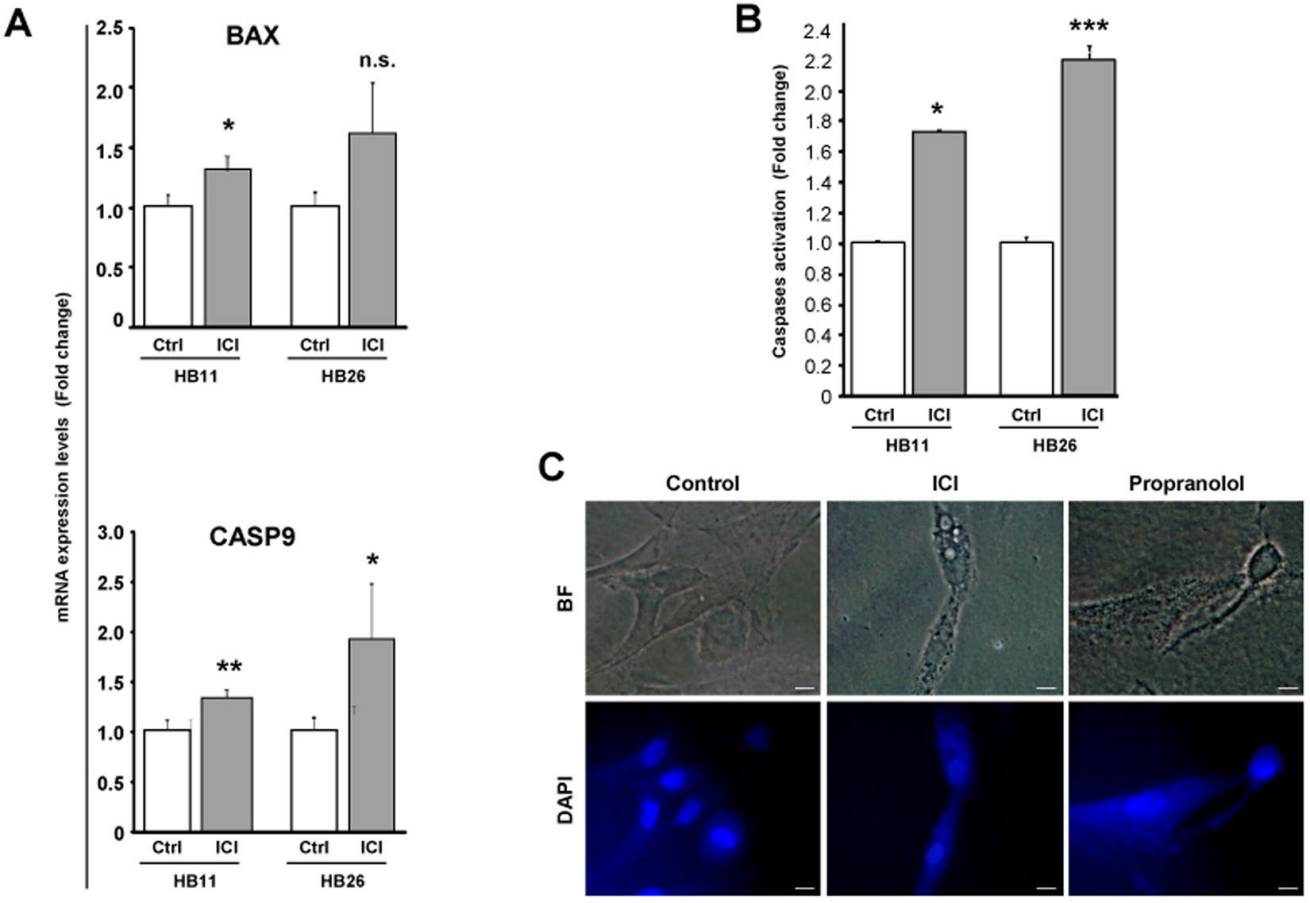
ICI-118,551 treatment induces cell apoptosis by stimulating pro-apoptotic genes Bax and Caspase 9. HB 11 (light grey) and HB26 (dark grey) primary cultures were incubated with 100 μM ICI for 72 h incubation. Relative mRNA expression levels of the apoptosis-related genes *BAX* and *CASP9*, measured by RT-qPCR. (**A**) Caspases 3&7 activity was quantified using the luminometric kit Caspase-Glo® 3/7 Assay. (**B**) HB18 cells were cultivated on coverslip in P-24 well plates, and treated with either 100 μM ICI or Propranolol for 24 or 48 h. Cells were fixed with PFA 4% for 30 min at 4 °C and washed with PBS. Coverslips were mounted in a drop of Pro-long DAPI mounting reagent (Life) and observed by fluorescence microscopy to determine nuclear integrity. (**C**) Representative pictures of 3 HB primary cell cultures. Scale bars represent 10 µm.

The decrease in viability of HB following ICI treatment is due to apoptosis as it happened for Propranolol^21^. This fact has been investigated by quantifying the expression of mRNA of two pro-apoptotic genes, *BAX* and the executor protease *Caspase 9*. Figure 2A shows how the expression of both genes is significantly upregulated (between 1.3–2 fold) after treatment with ICI in HBs, from different patients. On the other hand, apoptosis was also quantified measuring Caspase 3 and 7 activity by luminometry (Fig. 2B) following ICI treatment. A significant increment of caspase enzymatic activity of 1.7 fold was detected in treated versus untreated cells.

In order to obtain a visual confirmation of the apoptotic process on HB cells after Porpranolol or ICI treatment, nuclear integrity was assessed by DAPI staining. As shown in Fig. 2C, incubation for 48 hours with 100 μM Propranolol and ICI trigger the nuclear disassembly, supporting the genetic and molecular results shown in Fig. 2A,B.

### ICI-118,551 inhibits hemangiosphere formation from VHL tumour cells

**Figure 3:**
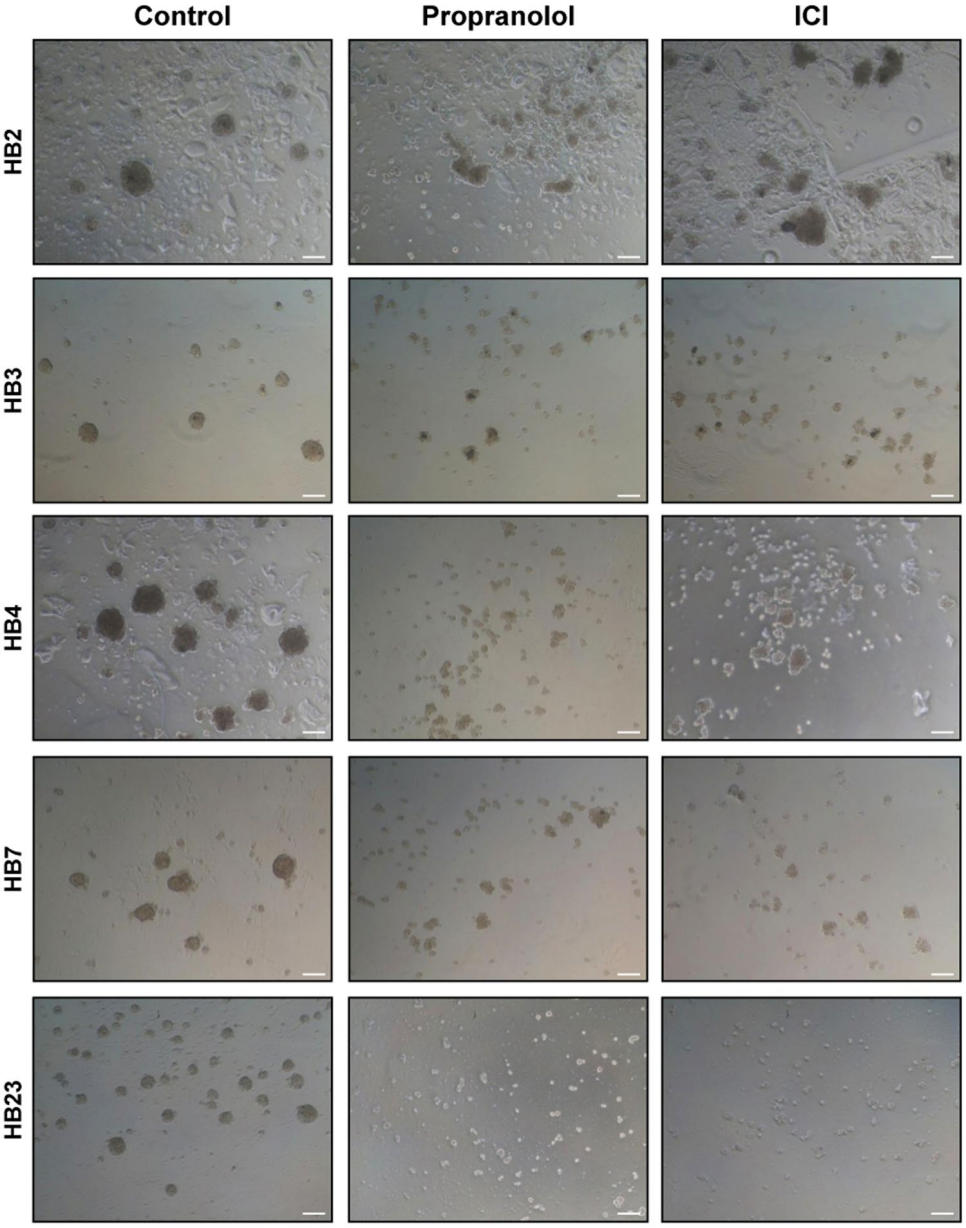
Propranolol and ICI-118,551 inhibit hemangiosphere formation from VHL patient cells. Six different HB primary cell cultures were treated for 7 days with either 100 μM Propranolol or ICI. Round sphere-like structures with a well-defined border appear as aggregates from VHL cell tumours. However, after the treatment, the spheres appear disaggregated and the observed groups are irregular with few cells. Pictures were taken the last day of treatment. Scale bars represent 100 µm.

Tumour cells, when cultured avoiding adherence, form organized and clearly defined border spheres around a core of cancer stem cells (CSC)^44,45^. Anti-tumoral agents show its properties by inhibiting or disrupting the formation of the spheres by disaggregation of cells. Figure 3 shows hemangiosphere cultures inhibition from 5 different HBs which were treated or not with 100 μM Propranolol or ICI for 7 days. As shown in Fig. 3, hemangiospheres are disrupted by β-blockers being especially conspicuous after ICI treatment on HB4 and HB23 cells.

### *In vitro* antiangiogenic properties of ICI-118,551

**Figure 4:**
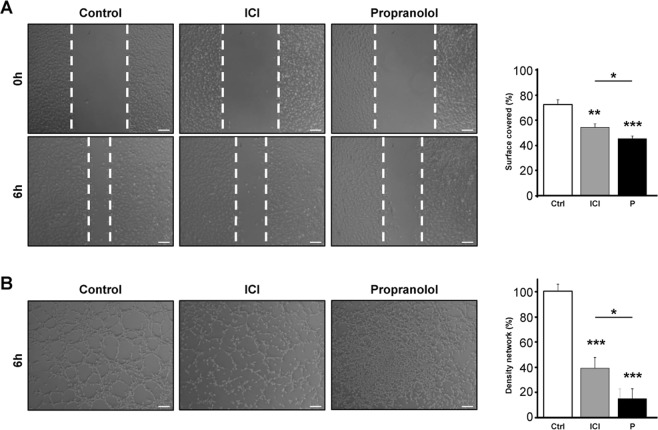
Propranolol and ICI-118,551 inhibit the angiogenic process (wound healing and tube formation assays) *in vitro*. (**A**). Shows representative images of scratch wound healing assay of HUVEC cells treated with either 100 μM ICI or Propranolol (P). Area covered after 6 hours was pictures and quantified showing that β-receptor blockage significantly impairs endothelial cell migration. (**B**) Shows representative images of tube formation assay of HUVEC cells treated with either 100 μM ICI (I) or Propranolol (P) for 6 hours. The number of closed cells (density network) was quantified as described in Material and Methods and averaged from 5 different fields in each condition. Density network quantification shows that Propranolol and ICI significantly inhibit the tube formation process. Data denote mean ± S.D. Scale bars represent 100 µm.

Angiogenesis is the property of formation of new vessels from pre-existing ones. Basically, the angiogenic process is based on the activation and further migration of the endothelial cells and tubulogenesis or formation of new vessels. These processes can be studied *in vitro* by the test of wound healing (Fig. 4A) and Matrigel tubulogenesis assay (Fig. 4B).

Confluent monolayers of HUVECs were disrupted by scratching their surface. This wound healing test was followed in time to monitor the migration of cells. Figure 4A shows how treatment with either 100 μM ICI or Propranolol delay the closure of the wound compared to control cells interfering with the migration process, as shown in the graph after quantification.

Figure 4B shows the characteristic tube network formed by endothelial cells on Matrigel after 6 hours, imitating the *in vivo* vessels network. However, if cells are pre-treated either with 100 μM ICI or Propranolol, the number of closed structures is dramatically decreased. The average quantification of closed tubules in five different optic fields is shown in Fig. 4B. Notably, Propranolol and ICI are acting as inhibitors of angiogenesis, migration, and of tubulogenesis processes.

### ICI-118,551 is an antiangiogenic agent acting by inhibiting HIF-target genes

**Figure 5:**
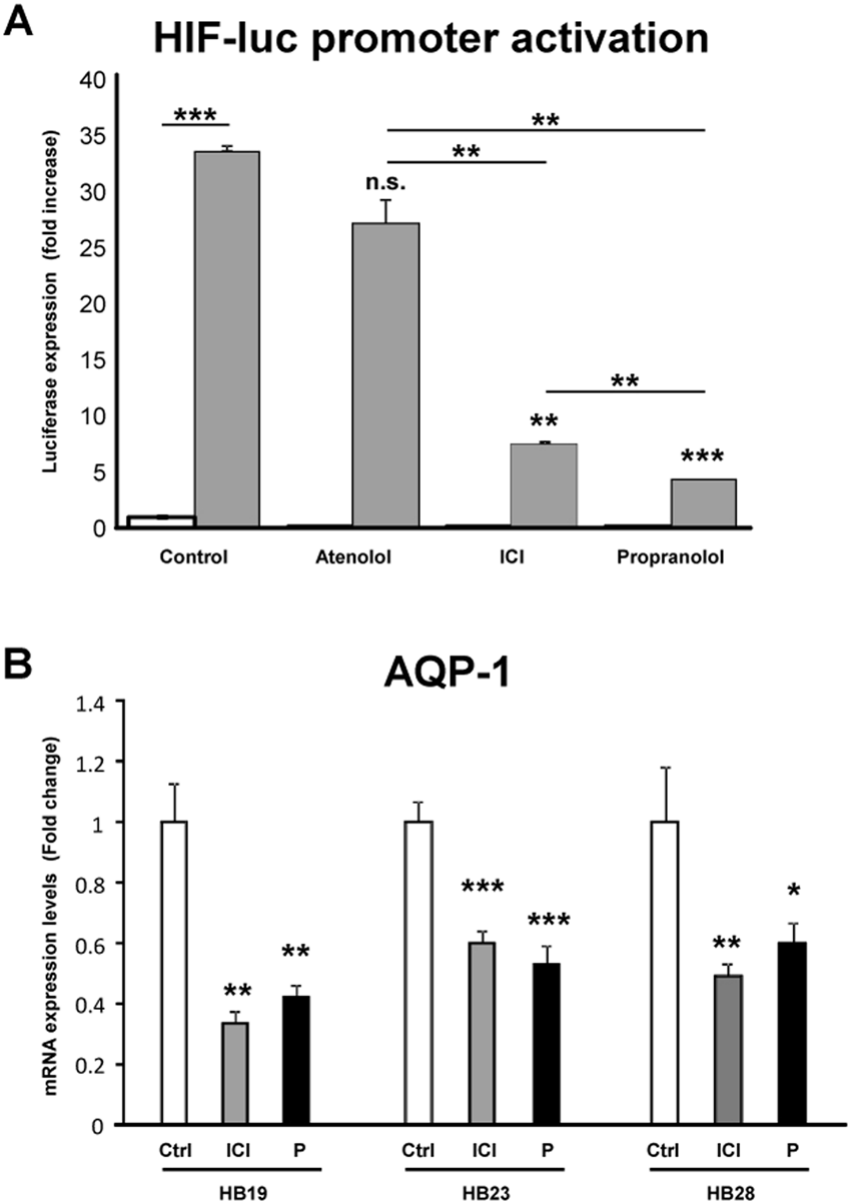
(**A**) Effect of β2-adrenergic receptor blockage on HIF targets. HeLa 9xHRE-Luc cells under normoxia (white bars) or hypoxia induced with 100 µM DFO (grey bars) were incubated with either 100 µM Atenolol, or ICI, or Propranolol for 24 hours. Then, luciferase-HIF dependent activity was measured. The bar histograms show the relative luciferase activity measured. Treatment with the β2-blockers Propranolol or ICI significantly inhibited the hypoxia stimulation induced by DFO. Data denote mean ± S.D (n = 3). (**B**) Propranolol and ICI, decrease the expression of the HIF target AQP-1. Different HB cells cultures (HB19, HB23, HB28) were treated with both β-blockers during 48 h and the expression of AQP-1 mRNA were measured by Real Time PCR. Propranolol and ICI significantly decrease the AQP-1 expression.

Propranolol and ICI are both able to block β2-adrenergic receptors. To test the hypothesis that by blocking the adrenergic β2-receptors, HIF transcriptional activity is inhibited, a system of luciferase reporter cell, HeLa 9xHRE-Luc, was used. To induce hypoxic conditions, HeLa 9xHRE-Luc cells were cultured with 100 μM Deferoxamine (DFO) and then treated with 100 μM Propranolol, ICI, or Atenolol for 48 hours. The luciferase activity was measured by luminometry. Figure 5A shows how luciferase expression is triggered under hypoxic conditions, and how this upregulation is dampened by Propranolol and ICI. Interestingly, there is some effect of Atenolol on the luciferase activity but this is negligible compared to the β2-antagonists (Propranolol and ICI). This result suggests that the decrease of HIF responsive transcription is mainly mediated through the β2-receptor blockade.

To test the impact of Propranolol and ICI on HIF-1α driven target genes, *Aquaporin 1 (AQP-1)* expression was studied by real time PCR. This target is of special interest in HBs, since *AQP-1* codes for a transmembrane protein water channel. *AQP-1* expression is induced by HIF-1α^46–48^. This enhanced expression may increase liquid flow across the cell membrane leading to cystic growth, common in VHL tumours. As shown in Fig. 5B *AQP-1* expression was significantly decreased in different HB cells cultures (HB19, HB23, HB28) treated with Propranolol or ICI.

### HIF-1α nuclear translocation is altered by the β-adrenergic receptor blockers

To unravel the possible molecular mechanism of action of Propranolol and ICI and to understand its effect on HIF target downregulation targets, HIF-1α cellular distribution was addressed by an immunofluorescence confocal assay. Blocking HIF-1α nuclear translocation should impair HIF targets, as described in cancer cells treated with Vorinostat^49^.

**Figure 6:**
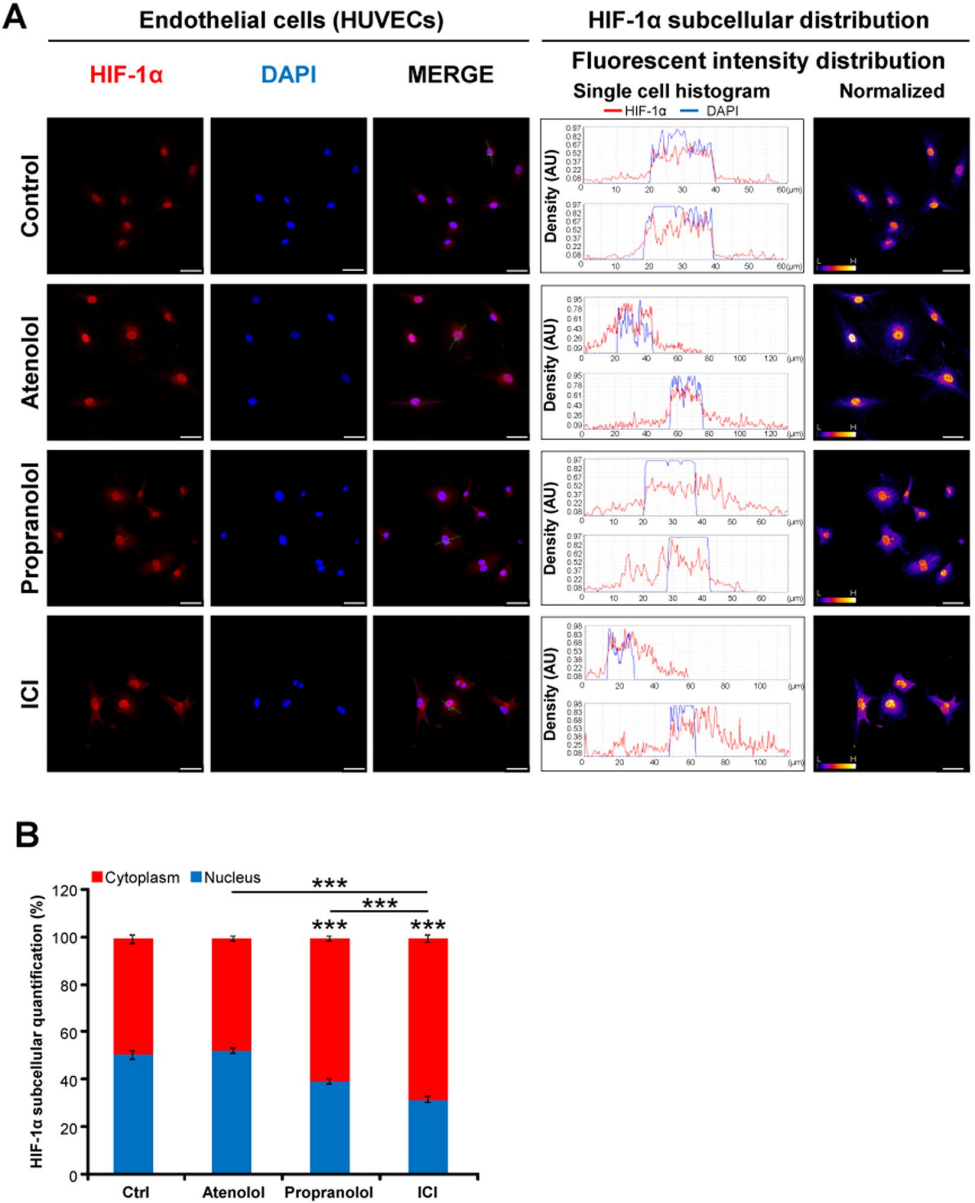
Effect of β2-adrenergic receptor blockage on HIF-1α subcellular distribution in endothelial cells. HUVEC cells under 100 µM DFO were incubated with either 100 µM Atenolol, or Propranolol, or ICI for 48 hours. Immunofluorescence detection and confocal microscopy processes are described in Material and Methods. (**A**) Shows representative images of the mouse anti-human HIF-1α antibody stains (red), DAPI nuclear staining (blue), and merged (middle column). Furthermore, the analysis of the distribution of HIF-1α alongside the cell is shown in two manners: i) as intensity histograms; were nuclei appear in blue (DAPI) and HIF-1α in red and ii) as a normalized distribution of HIF-1α intensity. (**B**) Shows the percent distribution of HIF-1α in the nuclei (blue) or cytoplasm (red). Treatment with the β2-antagonists Propranolol or ICI significantly inhibited the internalization of HIF-1α, being accumulated into the cytoplasm. Data denote mean ± S.D (n = 8–10). Scale bars represent 50 µm.

Firstly, as a proof of principle HUVEC cells under an hypoxic controlled condition with 100 µM DFO for 24 h, were treated with 100 µM of Atenolol, or Propranolol, or ICI, or vehicle. In this assay, HIF-1α was visualized and its nuclear or cytoplasmic localization (Fig. 6A) was measured in a quantitative manner (Fig. 6B). Figure 6A shows HIF-1α labelled with Alexa 568 (in red), nucleus with DAPI (in blue) and merged image, as well as cellular distribution histograms plus a normalized HIF-1α density distribution. In control or Atenolol treated HUVECs, HIF-1α is preferentially allocated in nuclei. However, treatment with Propranolol or ICI changed significantly its cellular distribution. The translocation of HIF-1α to the nucleus is somehow precluded since HIF-1α now is also found over the cytoplasm, as shown in Fig. 6A,B.

**Figure 7:**
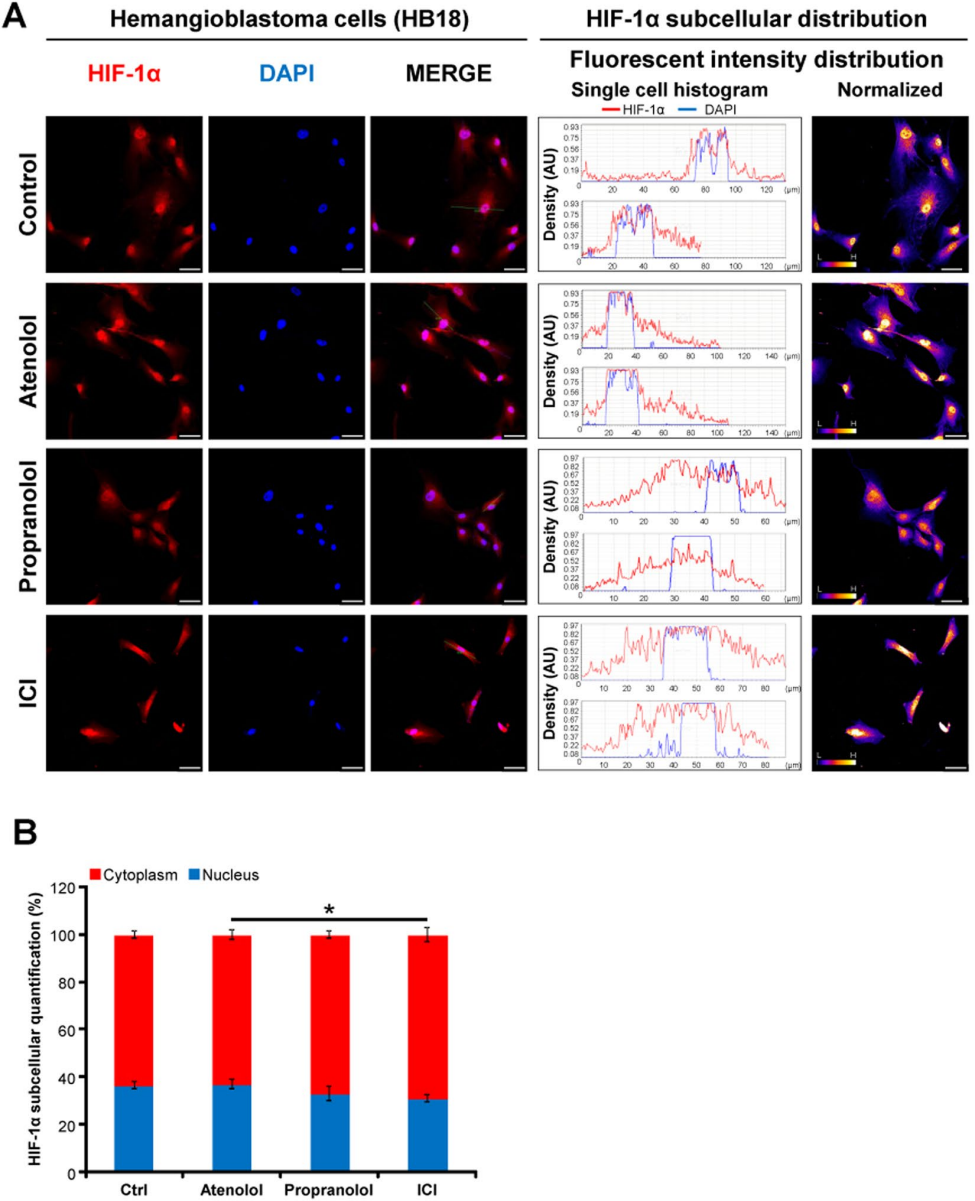
Effect of β2-adrenergic receptor blockage on HIF-1α subcellular distribution in HB primary culture cells. HB primary culture cells were incubated with either 100 µM Atenolol, or Propranolol, or ICI for 48 hours. Immunofluorescence detection and confocal microscopy processes are described in Material and Methods. (**A**) Shows representative images of the mouse anti-human HIF-1α antibody stains (red), DAPI nuclear staining (blue), and merged (middle column). Furthermore, the analysis of the distribution of HIF-1α alongside the cell is shown in two manners: i) as intensity histograms; were nuclei appear in blue (DAPI) and HIF-1α in red and ii) as a normalized distribution of HIF-1α intensity. (**B**) Shows the percent distribution of HIF-1α in the nuclei (blue) or cytoplasm (red). Treatment with the β2-blockers Propranolol or ICI significantly inhibited the internalization of HIF-1α, being accumulated into the cytoplasm. Data denote mean ± S.D (n = 8–10). Scale bars represent 50 µm.

Next, the same procedure was performed in HBs cultures. In untreated (Control) and Atenolol-treated HB cells, HIF-1α is translocated and therefore, is mainly localized inside the nucleus (Fig. 7A), although some staining is also visible in cytoplasm, due to the constitutively hypoxic physiology of HBs. The situation turns out completely different when Propranolol and ICI treatments are added. In these cases, HIF-1α is not as localized in nuclei as before. This situation can be clearly observed comparing the histograms shown in Fig. 7B, where the staining distribution was quantified by the ImageJ-FIJI. Notably, there is no significant difference between untreated and Atenolol treated-HB cells, and neither between Propranolol and ICI, indicating that the effect is mainly via β2-receptors.

In summary, the results of Figs 6 and 7 demonstrate that Propranolol and ICI, both β2-blockers are preventing in some way the translocation of HIF-1α to nuclei which may explain the decrease of gene expression of HIF-targets shown in Fig. 5 and was previously shown in Albiñana *et al*., 2015/2017^19,21^.

## Discussion

Most of rare diseases are not of primary interest for the industry due to their low incidence and the lack of benefits for the industry. The case of VHL disease, an hereditary rare cancer, maybe an exception due to its close connection with a bigger pathology like cancer.

VHL patients need several different CNS and retinal surgeries along their lifespan. Unluckily, there is an accumulative morbidity in neurologic and retinal functions after each surgery. After CNS HB surgical resection, only 20% of the patients maintain or improve their preoperative symptoms^50^.

Furthermore, the systemic therapy used for VHL Hemangioblastomas in CNS has been unsuccessful so far. Even the most recent clinical trial reported only 42% of response in renal and pancreatic tumours but no response in CNS tumours^14,51^.

In this scenario, where surgery and pharmacological therapies show limited recovery and response for CNS VHL disease, an urgent requirement for new and more effective drugs with reduced side effects for these patients are demanded.

Some previous studies from Shepard *et al*. and our group have demonstrated that Propranolol, a non-specific β1-and β2-blocker, was efficient on *in vitro* treatment of CNS Hemangioblastomas primary cultures^19,21,52^. Basically, Propranolol was acting in two ways: i) decreasing cell viability by triggering apoptosis and ii) inhibiting the expression of HIF-dependent targets in VHL HB cells, where HIF-1α degradation is impaired^21^. Moreover, a phase 3 clinical trial, conducted by the Spanish VHL Alliance, addressed the therapeutical benefits of Propranolol in VHL patients suffering from retina HBs (a different subpopulation of the CNS Hemangioblastomas), Over the time of the trial, retinal tumours did not grow and HB-derived exudates present in 2 different patients, disappeared completely between 3 and 6 months of treatment^19,20^.

Importantly, Propranolol side effects of hypotension and bradycardia were registered and make difficult to keep a sustained treatment, in particular in patients with tendency to hypotension. These cardio-specific side effects are due to Propranolol binding to β1-specific cardio-receptors. Therefore, the elucidation of whether the therapeutical effects from Propranolol are derived from the blockage of β1, β2 or both receptors was critical.

The lack of effect of Atenolol (a specific β1-receptor antagonist) on cell viability and HIF regulation on HBs cultures (Fig. 1B) or HeLa 9xHRE-Luc reporter cells (Fig. 5) gave us the clue to look for an almost specific β2-blocker. By finding this, we should avoid the undesired cardio specific effects while keeping the therapeutical effects related to β2-blockade.

The present work demonstrates that the selective β2-adrenergic receptor blocker ICI, has similar effects that Propranolol^20^ and in some tests shows improved results. In particular, as shown in results, ICI decreases viability, triggers apoptosis, inhibits hemangiosphere formation, cell migration, and angiogenesis processes, as well as decreases HIF levels and HIF-1 α nuclear translocation. Moreover, we have also assessed that ICI has a similar antitumoral activity as Propranolol by lowering cAMP levels^22,48^, as shown in Supplementary Fig. 1. Altogether, ICI is partially counteracting the HIF response, constitutively activated in VHL tumour cells.

It is particularly interesting the fact shown in Fig. 1 that ICI and Propranolol are specifically decreasing the viability of HB cells derived from VHL tumours, while at the same doses healthy endothelial primary cultures are much less affected. This is due to the fact that HB cells express more β2-receptors than HUVECs in this work (Fig. 1D). Thus, the effect of the β2-blockers is preferentially exerted on HB tumour cells, this becoming a very important issue for the specificity and safety of the treatment. On the other hand, the lack of effect of Atenolol on the viability of either HB or HUVECs proves that the decrease in viability is exerted through the two β2-antagonists, Propranolol and ICI (Fig. 1C).

An effect reported for the first time, to the best of our knowledge, is the ability of HB primary cultures, to form hemangiospheres. These spheres, observed currently in cancer cell lines from breast (MCF-7)^53^ or glioblastoma (U-87)^54^ are organized structures around at least one CSC in the core of the sphere. This proves that, although HBs are considered “non malignant tumours” they also contain some CSC, able to organize hemangiospheres. However, there may be less abundant CSC compared to other tumours, since hemangiospheres are smaller and show size variability *in vitro* among different HB primary cultures. One of the “standard” *in vitro* tests, for antitumoral therapeutic agents is the capacity to disrupt, the formation of these structures. Interestingly, as shown in Fig. 3, Propranolol and ICI inhibit hemangiosphere formation in HB cells derived from VHL patients, showing its potential therapeutical capabilities.

Moreover, as VHL is a pseudohypoxic syndrome, the inhibition by Propranolol and ICI of *in vitro* angiogenesis is highly relevant. As shown in Fig. 4, Propranolol and ICI inhibit the processes of cell migration and tubulogenesis both are necessary processes for angiogenesis. Angiogenesis is dependent on the activation of HIF program, constitutively active in VHL hemangioblastomas. We demonstrated in 2015 that Propranolol was able to inhibit the hypoxia inducible activity in HeLa 9xHRE-Luc cells. In this work we also show that ICI is acting as Propranolol, by precluding the hypoxia activation of HIF targets^21^. More important, since we are postulating that this effect is also dependent on the β2-blockade, Fig. 5 supports our hypothesis since the decrease in the HIF reporter activity is minimal after Atenolol treatment when compared to the effect of the β2-blockers Propranolol and ICI.

One important HIF target in VHL disease is AQP-1. Its expression was found to be involved not only in water transport but also in tumorigenesis, HBs become clinically manifest through the development of huge associated cysts^46,47^. As we can see in different HBs, the expression of AQP1 decreased significantly after Propranolol and ICI treatment.

Previously, we hypothesized a mechanism of action for Propranolol, where the HIF dependent HRE targets, VEGF, Sox2, Oct-4, Epo, and miR210, were clearly downregulated, also showing how Propranolol decreased the amount of HIF^19,21^. Trying to deepen into the mechanistic pathway, Figs 6 and 7 beautifully show how ICI treated cells have decreased amounts of HIF-1α in the nuclei.

In 2017, Propranolol was designated as orphan drug for the treatment of VHL by the European Medicines Agency. We propose ICI as a new therapeutic step forward, following the line of the β2-antagonists, but avoiding the cardio specific effects due to β1-binding as with Propranolol.

Taking into account Propranolol’s β1-2 antagonist activity and success in off-label clinical trials for rare cancer (melanoma, angiosarcoma, and metastasic paraganglioma), we think that ICI may show similar therapeutical properties for the same type of rare carcinomas, avoiding the β1 blocking vascular effect. In addition, tumours like clear cell renal carcinomas (ccRCC) where VHL is mutated arise as a potential target for β-blockers^55–58^.

Altogether, we conclude that ICI might be an interesting drug to have in the common pharmaceutical arsenal available to treat pseudohypoxic syndromes, in particular the VHL disease, without excluding other types of HIF-mediated diseases.

## Methods

### Cell culture and treatments

CNS HB primary cultures from excess of resected surgery pieces of VHL patients were obtained after the patients had provided written informed consent. HB primary cultures were purified as previously described^21^ and cultured in RPMI supplemented with 20% Fetal Bovine Serum (FBS), 2 mM L-glutamine, and 100 U/ml penicillin/streptomycin (all from GIBCO, Grand Island, NY, USA).

Primary human umbilical vein endothelial cells (HUVEC), were cultured in EGM-2 (Lonza, Walkersville, MD, USA) supplemented with 10% FBS, 2 mM L-glutamine, and 100 U/ml penicillin/streptomycin (GIBCO).

The human cervix adenocarcinoma HeLa cells (ATCC, CCL-2), stably transfected with a hypoxia reporter formed by nine copies in tandem of the hypoxia responsive element plus the luciferase gene (HeLa 9xHRE-Luc), were cultured in DMEM supplemented with 10% FBS; 2 mM L-glutamine, and 100 U/ml penicillin/streptomycin (GIBCO).

To induce hypoxic conditions, HUVEC and HeLa 9xHRE-Luc cells were treated with 100 μM desferrioxamine (DFO) (Sigma-Aldrich, San Luis, MO, US).

CNS Hemangioblastomas, HeLa 9xHRE-Luc, and HUVEC cells were incubated with different doses of β-adrenergic receptor blockers for the time and dose indicated on each experiment. Atenolol (Sigma), was dissolved in DMSO (Merck, Darmstadt, Germany), while Propranolol and ICI (Sigma) were dissolved in distilled water.

All the cellular assays were performed at 37 °C, 5% CO_2_ and humidity conditions.

### Cell viability assay

The viability of HB and HUVEC cells was measured by the Luminescent Cell Viability Assay (Promega, Madison, WI, USA). This is a homogeneous quantitative method to determine the number of viable cells in culture based on quantitation of the ATP presence, which indicates metabolically active cells. Briefly, 5 × 10^3^ cells were seeded in 96-well/plates and cultured in 100 μl with increasing doses [0–200 μM] of Atenolol, or Propranolol, or ICI for the time indicated for each experiment in results. Then, 100 μl/well of Cell Titer-Glo reagent (Lysis buffer, Ultra-Glo Recombinant Luciferase, Luciferine, and Mg^2+^) was added and gently mixed for 15 minutes at room temperature (RT). Next, luminescence was measured using a Glomax Multidetection System (Promega).

### Caspase activation assay

The Caspase-Glo® 3/7 Assay (Promega) is a luminescent assay that measures caspase-3 and caspase-7 activities. Luminescence is proportional to the amount of caspase activity presence. Briefly, 5 × 10^3^ cells were seeded in 96 well/plates and cultured in 100 μl with increasing doses of [0–200 μM] Atenolol, or Propranolol, or ICI for the time indicated for each experiment in results. Then, 100 μl/well of Caspase Glo® 3/7 Reagent (Lysis buffer, Ultra-Glo Recombinant Luciferase, DEVD-aminoluciferine, and Mg^2+^) was added and gently mixed for 1 hour at RT. Next, the luminescence was measured in a Glomax Multidetection System (Promega).

### Hemangiospheres formation and culture

To induce hemangiospheres formation, HB cells were grown in suspension in phenol red free DMEM:F-12 medium (GIBCO) supplemented with GlutaMAX (GIBCO), B27 Supplement 50X (GIBCO), 20 ng/ml EGF (Lonza, Basel, Switzerland), 20 ng/ml b FGF (Lonza), and 1% penicillin/streptomycin (GIBCO). 5 × 10^4^ cells/ml were plated at a density of on ultra-low attachment 75 cm^2^ cell culture flasks (Corning, Corning, NY, US). Cultures were treated or not with 100 μM Propranolol or ICI for 7 days.

### Wound healing and tube formation *in vitro* assays

Cell motility was assessed by wound healing recovery *in vitro* assay, in which a confluent monolayer of HUVECs in a 24-well plate was scratched by T-200 tip to make wounded gaps. Then, cell debris was washed out with PBS and fresh complete medium supplemented with PBS (as control), 100 µM Propranolol, or ICI for 6 hours. Endothelial cell migration into the denuded area was monitored at 0 and 6 hours post-wounding. Images were taken with an Olympus digital camera and ImageJ program (NIH, Bethesda, MD, US) was used to quantify the wound healing process.

For tube formation assays, 24 well-plates were previously coated with 100 μl standard Matrigel (BD Biosciences, Bedford, MA, USA) diluted 1:2 in serum-free DMEM plain medium (GIBCO). Then, 1 × 10^5^ HUVECs were seeded and cultured in fresh complete medium supplemented with PBS (as control), or with 100 µM Propranolol or ICI for 6 hours. Images were taken with an Olympus digital camera and quantification of connected tubules was performed using Adobe Photoshop CS3 software (San José, CA, US).

### Luciferase reporter assay

1 × 10^4^ HeLa 9xHRE-Luc cells were cultured in a 24 well-plate. On the next day, cells under normoxia (as control) or under hypoxia with 100 µM DFO were treated or not with 100 µM Atenolol, Propranolol, or ICI for 48 h. After the incubation, cell lysate luciferase activity was analyzed using the Luciferase reporter assay system (Promega) in a GloMax®-Multi Detection System (Promega), following the manufacturer’s instructions.

### Real-time RT-PCR

Total RNA was extracted from HB cells using Nucleo Spin RNA kit (Macherey-Nagel, Düren, Germany). One microgram of total RNA was reverse-transcribed in a final volume of 20 μl with the First Strand cDNA Synthesis Kit (Roche, Mannheim, Germany) using random primers. SYBR Green PCR system (BioRad, Hercules, CA, US) was used to carry out real-time PCR with an iQ5 system. Primers used for qPCR are: 18S Fwd: 5′-CTCAACACGGGAAACCTCAC-3′, 18S Rv: 5′-CGCTCCACCAACTAAGAACG-3′; BAX Fwd: 5′-CACTCCCGCCACAAAGAT-3′, BAX Rv: 5′-CAAGACCAGGGTGGTTGG-3′, CASP9 Fwd: 5′-CCCAAGCTCTTTTTCATCCA-3′, CASP9 Rv: 5′-TTACTGCCAGGGGACTCGT-3′, AQP1 Fwd: 5′-CCTCCCTGACTGGGAACTC-3′, AQP1 Rv: 5′-GGAGGGTCCCGATGATCT-3′.

### cAMP quantitative determination

The cAMP levels in HB were determined using the cAMP-Glo Assay (Promega). 5 × 10^3^ HB cells were seeded in 96 well/plates and cultured in the induction buffer: serum-free RPMI basal medium, 100 μM Ro 20-1724 (Sigma-Aldrich) and 500 μM IBMX (Sigma-Aldrich). The cells were then incubated with ICI or Propranolol for 30 minutes at 37 °C. Then, lysis buffer, cAMP reaction buffer, and cAMP detection solution were added according to the manufacturer’s specification. Finally, Kinase-Glo luminescent reagent was added and luminescence (cAMP activity) was measured in a Glomax luminometer (Promega).

### DAPI staining and fluorescence microscopy

The DAPI (4′,6-diamidino-2-phenylindole) staining method was used to detect the visual symptoms of apoptosis generated by ICI or Propranolol in HB cells.

Briefly, 5 × 103 HB cells were seeded on sterile, collagen-coated coverslips (13 mm diameter, VWR international, Radnor, PA, US) placed at the bottom of a 24 well-plate. On the next day, HB cells were treated with 100 µM Propranolol, or ICI for 24 and 48 h. Then, cells were washed with PBS and fixed with 3% PFA for 10 minutes at RT. After two PBS washing steps, samples were incubated mounted on glass slides using Prolong + DAPI mounting media (Molecular Probes, Eugene, OR, US). 40x confocal images were taken using the fluorescence confocal microscope Sp5 (DMI6000 CS Leica Microsystems, Wetzlar, Germany). FIJI-Image J software tool (NIH, MD, US) was used to identify and analyze the apoptotic nuclei.

### Immunofluorescence microscopy

Immunofluorescence analyses were performed to evaluate the effect of Atenolol, Propranolol, and ICI on translocation of HIF-1α from cytoplasm to the nucleus. 5 × 10^3^ HUVEC and HB cells were seeded on sterile, collagen-coated coverslips (VWR international) placed at the bottom of a 24 well-plate. On the next day, HB cells which have a constitutive hypoxic condition and HUVECs cells under 100 µM DFO were both treated with 100 µM Atenolol, or Propranolol, or ICI for 48 h.

Then, cells were washed with PBS and fixed with 3% PFA for 10 minutes at RT. After two PBS washing steps, samples were incubated with blocking solution (1% Goat Serum and 1% BSA in PBS) for 1 h at RT. Then, cells were incubated overnight at 4 °C with mouse anti-human HIF-1α antibody (1:100) (BD, Franklin Lakes, NJ, US.). Following this, cells were washed thoroughly four times with PBS and incubated for 1 h at RT with goat anti- Mouse IgG (H + L)-Alexa fluor 568 conjugate antibody (1:200) (Thermo Fisher Scientific, Waltham, MA, US). Finally, cells were washed with PBS and coverslips were mounted on glass slides using Prolong + DAPI mounting media (Molecular Probes). 40x confocal images were taken using the fluorescence confocal microscope Sp5 (Leica Microsystems). Red and blue channels represent HIF-1α and DAPI stains, respectively. FIJI-Image J software tool (NIH) and LAS AF Lite program from Leica, were used to identify and quantify the nuclear or cellular intensity of HIF-1α alongside the longer diameter of the selected cells.

### Statistical analysis

Results are presented as mean ± SEM. Statistical analyses were performed using the Student’s t-test. Statistical significance was defined when P < 0.05 (*P < 0.05; **P < 0.01, ***P < 0.001).

### Ethical issues

All methods described in the present manuscript were carried out in accordance with all the international relevant guidelines and regulations of obliged fulfilment in the National Research Council of Spain (CSIC), and in agreement with worldwide regulations for Molecular Research. The Ethical committee of CSIC approved the protocols and procedures of the projects SAF 2014 52374-R and SAF2017-83351R, framework and funding for the experiments presented.

Informed consent was obtained from all subjects or, if subjects are under 18, from a parent and/or legal guardian.

## Supplementary information

Supplementary Info
