# Supplementary material for: The β2-adrenergic receptor antagonist ICI-118,551 blocks the constitutively activated HIF signalling in hemangioblastomas from von Hippel-Lindau disease
Source: Sci Rep. 2019 Jul 11;9:10062. doi: 10.1038/s41598-019-46448-6 (PMC6624208; doi:10.1038/s41598-019-46448-6)
Supplement: Supplementary file 1 — Supplementary Info [file 41598_2019_46448_MOESM1_ESM.pdf]

TITLE

The β2-adrenergic receptor antagonist ICI-118,551 blocks the constitutively activated HIF signalling in hemangioblastomas from von Hippel-Lindau disease.

Cuesta AM[1#], Albiñana V[1#], Gallardo-Vara E[1#], Recio-Poveda L[1], Isabel de Rojas-P[1], Villar Gómez de Las Heras K[2], Aguirre DT[3], Botella LM[1]*.

AUTHOR INFORMATION

[1] Centro de Investigaciones Biológicas, Consejo Superior de Investigaciones Científicas (CSIC), and Centro de Investigación Biomédica en Red de Enfermedades Raras (CIBERER), group U707, 28040 Madrid, Spain.

[2] SSCC del Servicio de Salud de Castilla-La Mancha (SESCAM), Toledo, Spain.

[3] Department of Neurosurgery, Fundación Jiménez Díaz (IIS-FJD), Av. Reyes Católicos, 28040, Madrid, Spain.

E-mail addresses:

Cuesta AM (cuestaam@gmail.com); Albiñana V (vir_albi_di@yahoo.es); Gallardo-Vara E (eunate.gallardo@yale.edu); Recio-Poveda L (luciarecio@hotmail.com); Isabel de Rojas-P (iderojas@ucm.es); Villar Gómez de Las Heras K (kvillar@jccm.es); Aguirre DT (dtaguirre@fjd.es); Botella LM (cibluisa@cib.csic.es).

[#]Cuesta AM, Albiñana V and Gallardo-Vara E contributed equally to this work.

*Corresponding author: Luisa Maria Botella.

Centro de Investigaciones Biológicas, Consejo Superior de Investigaciones Científicas (CSIC), and group U707, Centro de Investigación Biomédica en Red de Enfermedades Raras (CIBERER), 28040 Madrid, Spain. Phone: +34 91 837 31 12 (ext. 4312). FAX: +34 91 536 04 32. E-mail: cibluisa@cib.csic.es

Supplementary Table 1: Review on the main clinical trials using ICI-118,551.

Article	Condition measured (field of study)	Number of subjects treated	Subjects' condition. (average age)	Treatment dosage	Length of treatment	Conclusions and observations
1	Tachycardia (Cardiology)	12	Healthy population. (23.2)	5 mg<br>10 mg<br>20 mg<br>40 mg<br>80 mg	Single dosis (2 hours before exercise or infusions of isoprenaline sulphate)	- Significant changes on β2-AR mediated responses: diastolic, blood pressure, forearm blood flow, finger tremor and metabolic parameters.<br>- ICI selectively and competitively antagonizes β2-AR in humans.
2	Hypertension (Cardiology)	9	Hypertensive males (neither cardiac nor renal disease). (35)	50 mg every 8 hours (150 mg/day)	1 week	- ICI lowers blood pressure less than Propranolol and Atenolol.
3	Essential Tremor (Neurology)	10	Adult male patients. (40.7)	150 mg/day	1 week	- ICI may be useful in the management of essential tremor while having minor cardiovascular side-effects than non-selective β-AR antagonists.
4	Hypertension (Cardiology)	10	Previously diagnosed essential hypertension. (46)	50 mg every 8 hours (150 mg/day)	1 week	- No effect on blood pressure.<br>- Hypertensive patients don't respond to ICI while they do respond to Atenolol and Propranolol.
5	Heart Rate Variability (Cardiology)	17	Healthy males. (30)	25 mg	Single dosis (at sleeping time)	- No significant effect on heart rate.<br>- Salbutamol increases rate (β2 agonist) and ICI counteracts the effect.
6	Cardiovascular effects (Cardiology)	5	Healthy males. (30.6)	10 mg<br>20 mg<br>50 mg<br>100 mg	Single dosis 1.5-2h prior to tests	- At doses of 50 mg and over, ICI shows a significant effect on systolic interval time and systolic pressure and effect on β1-AR.
7	Anxiety, (Psychiatry)	17	Previously diagnosed anxiety disorder. (37.1)	50 mg every 8 hours (150 mg/day)	4 weeks	- No significant change in blood pressure or heart rate.<br>- No effect of ICI in anxiety control.
8	Flight Phobia Stress (Psychiatry)	12	Previously diagnosed flight phobia. (range 21-54)	50 mg every 8 hours (150 mg/day)	Hours before and during flight	- No significant changes in psychological stress.<br>- No beneficial effect on flight phobia.
9	Heart Rate Variability (Cardiology)	8	Healthy males. (23)	25 mg/night	1 week	- No significant effect on heart rate variability.

Supplementary References:

1- Arnold JM, O'Connor PC, Riddell JG, Harron DW, Shanks RG, McDevitt DG. Effects of the beta 2-adrenoceptor antagonist ICI 118,551 on exercise tachycardia and isoprenaline-induced beta-adrenoceptor responses in man. Br J Clin Pharmacol. May;19(5):619-30 (1985).

2- Vincent HH, Man in 't Veld AJ, Boomsma F, Derkx FH, Schalekamp MA. Is beta 1-antagonism essential for the antihypertensive action of beta-blockers? Hypertension. Feb;9(2):198-203 (1987).

3- Jefferson D, Wharrad HJ, Birmingham AT, Patrick JM. The comparative effects of ICI 118551 and propranolol on essential tremor. Br J Clin Pharmacol. Dec;24(6):729-34 (1987).

4- Robb OJ, Webster J, Petrie JC, Harry JD, Young J. Effects of the beta 2-adrenoceptor antagonist ICI 118,551 on blood pressure in hypertensive patients known to respond to beta 1-adrenoceptor antagonists. Br J Clin Pharmacol. Apr;25(4):433-8 (1988).

5- Silke B, Riddell JG. Evaluation of the effect on heart rate variability of some agents acting at the beta-adrenoceptor using nonlinear scatterplot and sequence methods. Cardiovasc Drugs Ther. Oct;12(5):439-48 (1988).

6- Harry JD, Norris SC, Percival GC, Young J. The dose in humans at which ICI 118,551 (a selective beta 2-adrenoceptor blocking agent) demonstrates blockade of beta 1-adrenoceptors. Clin Pharmacol Ther. May;43(5):492-8 (1988).

7- Cooper SJ, Kelly CB, McGilloway S, Gilliland A. Beta 2-adrenoceptor antagonism in anxiety. Eur Neuropsychopharmacol. Nov;1(1):75-7 (1990).

8- Ekeberg O, Kjeldsen SE, Eide IK, Greenwood DT, Enger E. Effects of beta 1- and beta 2-blockade on blood pressure and sympathetic responses to flight phobia stress. Clin Pharmacol Ther. May;47(5):599-607 (1990).

9- Silke B, Hanratty CG, Riddell JG. Heart-rate variability effects of beta-adrenoceptor agonists (xamoterol, prenalterol, and salbutamol) assessed nonlinearly with scatterplots and sequence methods. J Cardiovasc Pharmacol. Jun;33(6):859-67 (1999).

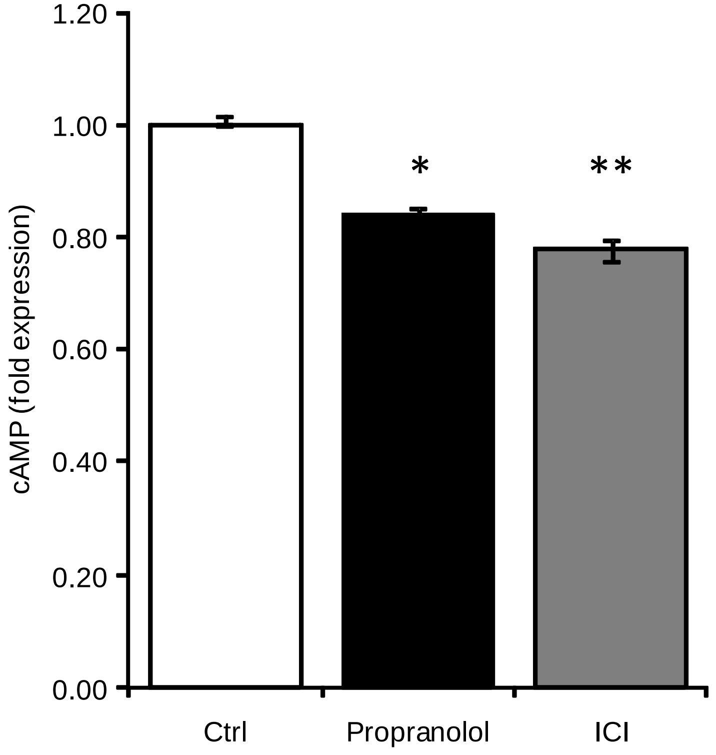

<u>Supplementary Figure 1:</u> cAMP levels of HB cells after vehicule, Propranolol and ICI-118,551 treatment. HB18 cells were incubated in the absence or presence of 100µM of both β-blockers for 30 minutes. The cells were lysed and the content of cAMP was measured following the manufactured instruccions cAMP- Glo Assay(Promega). The luminometry was quantified in Glomax Multi detteccion system. The cAMP levels decreased at 18% and 25% for propranolol and ICI, respectively. Differences were significant in both cases. Each condition is the mean of 6 replicates. The experiment was repeated 3 times.
